# The usefulness of metaphase I oocytes in women who undergo controlled ovarian hyperstimulation for intracytoplasmic sperm injection

**DOI:** 10.5935/1518-0557.20200062

**Published:** 2021

**Authors:** João Paolo Bilibio, Pânila Longhi Lorenzzoni, Arivaldo José Conceição Meireles, Yasmin Maciel, Pablo Sales, Fábio Costa do Nascimento

**Affiliations:** 1 Department of Obstetrics and Gynecology, Universidade Federal do Pará, Belém, Pará, Brazil; 2 Clínica de Reprodução Assistida Pronatus, Belém, PA, Brazil; 3 Grupo de Pesquisa Bilibio, Universidade Federal do Pará, Belém, PA, Brazil; 4 Programa de Pós Graduação de Ciências Médicas da Universidade Federal do Rio Grande do Sul, Porto Alegre, Rio Grande do Sul, Brazil.

**Keywords:** blastocyst, metaphase I, ICSI, IVF, oocyte

## Abstract

**Objective:**

The aim of this study was to evaluate the fertilization and blastocyst formation rates of oocytes in metaphase I (MI) obtained from women who underwent controlled ovarian hyperstimulation (COH) for intracytoplasmic injection.

**Methods:**

A prospective cohort study that included women from whom at least 1 MI and 1 MII oocyte were obtained after COH was performed. We collected 1,907 oocytes from 164 women (1291 MII, 352 MI and 258 prophase I or atretic). After oocyte classification, the MII and MI oocytes were incubated for 4 hours.

**Results:**

After 4 hours, the rescue maturation rate was 57.2%; 205 MI oocytes matured to MII oocytes *in vitro* (rescued MI-MII group), and 153 remained in MI (arrested MI group). The normal fertilization rates were directly associated with oocyte maturation, with rates of 79.1%, 60.2%, and 31.9% in MII, MI-MII and MI oocytes, respectively (*p*<0.001). Group arrested MI had an odds ratio (OR) of 7.6 (CI 5.2 - 11.2, *p*<0.001) for abnormal fertilization compared with Group MII. The blastocyst formation rate was directly associated with oocyte maturation, at 36.4% for MII, 11.4% for MI-MII and 0.6% for MI.

**Conclusion:**

Oocytes collected at the MI stage after OCH that did not mature to MII after rescue maturation had a blastocyst formation rate of only 0.6%, while those in MII and MI-MII had rates of 36.4% and 11.4%, respectively. However, we found a pregnancy with the birth of a healthy baby from a blastocyst formed after intracytoplasmic sperm injection (ICSI) of an MI oocyte.

## INTRODUCTION

Due to the increasing number of couples seeking to conceive via assisted reproduction technology, particularly in cases of *in vitro* fertilization (IVF), and due to the increasing number of women over 40 years of age who desire a new or first pregnancy, specialists have sought to identify the strategy that yields the best outcome (Huang & Rosenwaks, 2014). Controlled ovarian hyperstimulation (COH) was adopted to increase pregnancy rates. With COH, multiple follicles can develop. This yields multiple oocytes that can be aspirated and fertilized and enables the selection of the best embryos for embryonic transfer (Ingerslev *et al*., 2001). However, the ovarian response to stimulation is variable and depends mainly on each woman's ovarian reserve (Huddleston *et al*., 2010). With increasing maternal age, the quantity and quality of obtainable oocytes decreases; thus, age is considered the main influencer of ovarian response and oocyte quality and maturation (Su *et al*., 2017). IVF can overcome many causes of infertility, but it cannot reverse fertility decline in older women (Baker *et al*., 2010; Malizia *et al*., 2009).

The unresponsiveness of some oocytes to ovarian stimulation has not yet been explained, but it is possible that the different stages of follicle development yield oocytes with varying degrees of maturation after stimulation. During aspiration, oocytes are collected from heterogeneous groups of follicles, and mature oocytes in metaphase II (MII) or meiotically immature oocytes in metaphase I (MI) or prophase I (PI) may be captured (Stouffer & Zelinski-Wooten, 2004). The proportion of immature oocytes among collected oocytes generally ranges from 10 to 20%, and a high number of immature oocytes can negatively affect the ICSI outcomes of mature oocytes from the same woman (Braga *et al*., 2020).

Follicular maturation is extremely important for the generation of viable embryos for implantation and, consequently, for the success of IVF. To be fertilized, oocytes should ideally reach maturity, as oocytes in PI and MI are too morphologically immature to be fertilized for the formation of good-quality embryos. MI-stage oocytes can spontaneously undergo in vitro meiotic maturation to MII-stage oocytes within a few hours after collection (Strassburger *et al*., 2004), but they generally remain unused because their rates of fertilization, blastocyst formation and implantation are lower than those of MII oocytes that mature *in vivo* (Strassburger *et al*., 2004; Alcoba *et al*., 2015).

The pregnancy rate is directly associated to the number of embryos in the blastocyst stage that are formed during the IVF cycle, and this number depends on the quantity and quality of MII oocytes collected (Ben-Nagi *et al*., 2019). Since the number of these oocytes is inversely proportional to the age of the patient, the use of MI oocytes for IVF, especially for couples of advanced age, can be an alternative. Thus, when a small number of aspirated MII oocytes are available due to the patient's low response to ovarian stimulation, the use of *in vitro*-matured MI oocytes can be considered (Braga *et al.*, 2010).

The aim of this study was to evaluate the fertilization and blastocyst formation rates of oocytes in MI from women who underwent COH for intracytoplasmic injection (ICSI).

## MATERIALS AND METHODS

A prospective cohort study was performed among women who underwent COH for intracytoplasmic sperm injection (ICSI) during the study period, June 2018 to June 2019, at the Pronatus Reproductive Medicine Center, Belém, Pará State, Brazil. The National Ethics and Research Committee on Human Beings and by the Ethics Committee of Institute of Health Sciences, Federal University of Pará under protocol number CAEE 55625115.0.0000.0018 (institutional review board equivalent), approved the study.

The study included women from whom at least one MI oocyte and one MII oocyte was harvested after COH. As the objective of the study was to evaluate oocyte quality through fertilization rates and blastocyst formation rates, the exclusion criteria were having had chemotherapy, radiotherapy, or severe male factor (sperm concentration < 5 million /ml after seminal processing with the swim-up method).

The probable causes of infertility were assessed for all couples and were as follows: tubal factor (defined as an abnormality by hysterosalpingography or videolaparoscopy); male factor (defined as a sperm count less than 15 million/ml and excluding those with less than 5 million/ml after seminal processing with the swim-up method); low ovarian reserve (defined as <5 antral follicles evaluated by transvaginal ultrasound performed on the 2^nd^ or 3^rd^ day of the menstrual cycle); repeat abortion (two or more consecutive abortions); polycystic ovary syndrome; endometriosis (presence of endometriosis on videolaparoscopy or endometrioma on imaging studies); and undetermined cause.

The ovarian stimulation protocol began on the second day of the menstrual cycle after transvaginal ultrasound was performed to determine the antral follicle count; recombinant follicle-stimulating hormone (FSH, Elonva 150®, Organon) was applied subcutaneously. After day 6 of application, transvaginal ultrasound was performed every two days to monitor follicle development. When one of the largest follicles reached 14mm, gonadotrophin-releasing hormone (GnRH) antagonist (Orgalutran®, Organon) was administered daily, subcutaneously, until the use of recombinant human chorionic gonadotropin (rhCG). After day 9 of induction, recombinant FSH (Puregon®, Organon) at a dose of 150 IU per day was used until rhCG administration. When three or more follicles reached 17mm in diameter, oocyte maturation was stimulated with rhCG (Ovidrel 250mcg, Serono). Transvaginal ultrasound-guided oocyte recovery was performed 35 hours after hCG application.

Oocytes located in the follicular fluid, together with the cumulus cells, were collected and transferred to a plate containing HAM buffered culture medium (Modified HAM's F10, Irvine Scientific) and mineral oil (Light Oil - Irvine Scientific). After screening, all oocytes were subjected to a pure 100µL hyaluronidase solution (Ingase-hyaluronidase enzyme solution, 80IU/ml, Ingamed) for 30 seconds to remove cumulus cells. Then, all oocytes were transferred to a plate with buffered culture medium and supplemented with complete multipurpose handling medium (MHM-c, Irvine Scientific), covered with mineral oil for mechanical removal of corona cells. After total removal of corona cells, we classified the oocyte maturation with a magnifying glass, according to the presence of the first polar corpuscle (MII), the absence of the polar corpuscle (MI) and presence of germinal vesicles (prophase I - PI), rupture or atresia. A total of 1,907 oocytes from 164 patients were included for evaluation, and after denudation, there were 1,291 MII, 352 MI and 175 PI and 83 atretic follicles. For this study, the evaluation of oocyte maturity, the same senior embryologist from the team performed ICSI and embryo culture to blastocyst, and the image capture of each evaluation was carried out for further discussion if necessary.

After oocyte classification and counting, we transferred only those classified as MII and MI to a plate with a balanced single-step embryonic culture medium (Global Total), where we incubated them after denudation for up to 4 hours. We set a maximum time of 4 hours as the incubation limit for stabilization of mature MII oocytes and the rescued *in vitro*-maturation time for immature MI prior to ICSI.

After 4 hours in the single-step balanced embryonic culture medium (Global Total), 1,649 oocytes were fertilized by ICSI; 1,291 were in the MII stage (MII group). Of the 358 oocytes in the MI stage, 205 were rescued in vitro-matured MII oocytes (rescued MI-MII group), and 153 remained in stage MI (arrested MI). To proceed with the ICSI, we collected semen samples on the same day of the oocyte recovery process with sexual abstinence of up to a maximum of 5 days. We prepared the sperm by means of the swim-up procedure, and performed embryonic culture in a Thermo Scientific CO_2_ Incubator.

After 17 hours of ICSI, we assessed the fertilization rates of the MII, MI-MII and MI groups (we considered it as an adequate fertilization when the embryo had 2 pronuclei (PN), and we considered it a fertilization failure when the embryo had 1PN or ≥ 3PN).

On days 5 and 6, we assessed the rates of blastocyst formation in the MII, MI-MII and MI groups, and we considered only blastocysts that were suitable for fresh transfer or cryopreservation. The parameters for the blastocyst assessment were as follows: blastocyst classification according to the developmental stage (1: initial, 2: blastocyst, 3: expanded, 4: hatched/hatching), internal cell mass (A: prominent, B: easily discernible, C: difficult to distinguish), and trophectoderm (A: many cells forming a cohesive epithelium, B: few cells forming a loose epithelium, C: few cells).

Continuous variables with a normal distribution and equal variances were evaluated using ANOVA for independent samples. We used the chi-squared test for categorical variables, and a logistic regression model to determine the associations between the characteristics studied using the Pearson and chi-square correlations and calculated according to Sheskin. We calculated the odds ratio and 95% confidence interval according to Altman. The threshold for statistical significance was 5%. We ran the statistical tests using the Statistical Package for the Social Sciences 20 (SPSS Inc., Chicago, IL, USA) and MedCalc. Our results showed sufficient power, with the results of Fisher’s exact test and a mid-P test between 99.98% and 100%, to analyze the fertilization rate and blastocyst formation rate. Therefore, our findings are robust and valuable, when considering a power greater than 80% to be adequate.

## RESULTS

A total of 1,649 oocytes were subjected to ICSI and divided into the following 3 groups: 1,291 MII-stage oocytes in the MII group, 205 rescue in vitro-matured oocytes in the MI-MII group and 153 MI-stage arrested oocytes in the MI group. The *in vitro* maturation rate was 57.2%. The clinical and oocyte characteristics of the study cohort are shown in Table 1.

**Table 1:** Clinical and oocyte characteristics of the study group

Clinical and oocyte characteristics	Mean (SD)*
Maternal age (years)	35.6 (4.2)
Infertility time (years)	5.6 (5.1)
Menarche (years)	12.0 (1.5)
Days of induction (N)	10.3 (1.5)
Endometrial thickness (mm)	10.2 (2.1)
Days of sexual abstinence before semen collection	3.3 (1.9)
Total oocytes collected % (N)	100% (1907)
MII)^ϯ^	67.7% (1291)
MI)^ŧ^ total	18.7% (358)
[Arrested MI)^ŧ^ oocytes after 4 hours of incubation]	[8.0% (153)]
[Rescued MI-MII^§^ oocytes after 4 hours of incubation]	[10.7% (205)]
[Rescue maturation rate]	[57.2%]
Prophase I	9.1% (175)
Atresic	4.3% (83)

* SD: standard deviation

ϯ MII: metaphase II

ŧ MI: metaphase II

§ MI-MII: matured from MI to MI after 4 hours of incubation

The comparison of the normal fertilization rates among the MII, MI-MII and MI groups is shown in Table 2. The normal fertilization rate was directly related to oocyte maturation: the normal fertilization rates of the MII, MI-MII, and MI groups were 79.1%, 60.2% and 31.9%, respectively (*p*<0.001). MI oocytes had an OR of 7.6 (CI 5.2 - 11.2, *p*<0.001) for abnormal fertilization compared with MII oocytes and an OR of 4.0 (CI 2.5 - 6.3, *p*<0.001) compared with MI-MII oocytes. In addition, MI-MII oocytes had an OR of 1.9 (CI 1.4 - 2.6, *p*<0.001) for abnormal fertilization compared with MII oocytes.

**Table 2:** Comparison of the normal fertilization rates of the MII, rescued MI-MII and arrested MI groups

Stage of oocyte maturation	Total oocytes	Normal fertilization 2PN % (N)	Abnormal Fertilization % (N)	OR (95% CI)*	*p*-value^ϯ^
**MII**	1291	79.1% (956)	20.9% (335)		
**Rescued MI-MII**	205	60.2% (123)	39.8% (82)	1.9 (1.4 - 2.6)^ŧ^	<0.001^ŧ^
**Arrested MI**	153	31.9% (39)	68.1% (104)	7.6 (5.2 - 11.2)^||^	<0.001^||^
				4.0 (2.5 - 6.3)^§^	<0.001^§^

PN- pronucleus

* OR: odds ratio calculated according to Altman; CI: confidence interval 95%.

ϯ Pearson correlation and Chi-square calculation according to Sheskin

ŧ Comparison of MII-MI to MII

|| Comparison of MI to MII

§ Comparison of MI to MI-MII

The embryo polyploidy rate (≥ 3PN) for the stage of oocyte maturation is shown in Table 3. The risk of embryo polyploidy was higher in the MI group, with an OR of 2.6 (CI 1.28-5.41, *p*=0.008), than in the MII group. There was no increase in the risk of MI-MII *versus* MII oocyte polyploidy.

**Table 3:** Embryo polyploidy rate (≥ 3PN) for the different stages of oocyte maturation

Stage of oocyte maturation	Total oocytes	≥ 3PN (%)	OR (95% CI)*	*p*-value^ϯ^
**MII**	1291	3.55%	-	-
**Rescued MI-MII**	205	5.47%	1.6 (0.8 - 3.1)^ŧ^	0.195^ŧ^
**Arrested MI**	153	8.84%	2.6 (1.3 - 5.4)^§^	0.008^§^

PN- pronucleus

* OR: odds ratio calculated according to Altman; 95% CI: 95% confidence interval.

ϯ Pearson correlation and chi-square calculation according to Sheskin

ŧ Comparison of MII-MI to MII

§ Comparison of MI to MII

The blastocyst formation rate according to oocyte maturation is shown in Table 4. The blastocyst formation rate decreased as oocyte maturation decreased, with blastocyst formation rates of 36.4%, 11.4% and 0.6% in the MII, MI-MII, and MI groups, respectively. MI-MII oocytes had an OR of 4.5 (CI 2.9 - 7.1, *p*<0.001) for not forming a blastocyst compared with MII oocytes. In addition, MI oocytes had an OR of 86.9 (CI 12.1 - 623.4, *p*<0.001) for not forming a blastocyst compared with MI oocytes and an OR of 19.2 (IC 2.5 - 143.8, *p*=0.004) for not forming a blastocyst compared with MI-MII oocytes. Although the rate of blastocyst formation from the MI oocytes was extremely low, one blastocyst formed and led to a pregnancy that resulted in the birth of a healthy baby with a normal karyotype.

**Table 4:** Blastocyst formation rate according to oocyte maturation

Stage of oocyte maturation	Total oocytes	Blastocysts % N	D5 % N	D6 % N	OR (95% CI)*	*p*-value^ϯ^
**MII**	1201	36.4% (437)	74.5% (326)	25.5% (112)	-	-
**Rescued MI-MII**	205	11.4% (23)	69.6% (16)	30.4% (7)	4.5 (2.9 - 7.1)^ŧ^	<0.001^ŧ^
**Arrested MI**	153	0.6% (1)	0.0% (0)	100.0% (1)	86.9 (12.1 - 623.4)^||^	<0.001^||^
					19.2 (2.5 - 143.8)^§^	0.004^§^

D5 and D6 (blastocyst formation day)

* OR: odds ratio calculation according to Altman; 95% CI: 95% confidence interval.

ϯ Pearson correlation and Chi-square calculation according to Sheskin

ŧ Comparison of MII-MI to MII

|| Comparison of MI to MII

§ Comparison of MI to MI-MII

The proportions of oocytes collected at each maturation stage according to female age group are shown in Table 5. We found no differences in the maturation rates of oocytes collected from the different age groups evaluated.

**Table 5:** Rate of oocytes collected in each maturation stage according to the female age group

Maturation stage of oocyte	Oocyte maturation rate (%)			
	≤ 34 years	35-39 years	≥ 40 years	*p*-value*
**MII**	78.2%	76.0%	77.6%	0.652
**Rescued MI-MII**	11.0%	13.7%	16.4%	0.428
**Arrested MI**	9.2%	10.3%	6.6%	0.390

* Kendall correlation

The blastocyst formation rates according to female age group are shown in Table 6. There was a decrease in the rate of blastocyst formation from MII oocytes among women over 40 years of age (28.6% *versus* 37.5% and 38.4% for women over 40 years versus women 35 to 39 years and under 34 years, respectively, *p*=0.030). We found no differences in the rates of formation of blastocysts from MI-MII oocytes among the age groups evaluated.

**Table 6:** Blastocyst formation rate according to the female age group

	Blastocyst formation rate			
Maturation stage of oocyte	≤ 34 years	35-39 years	≥ 40 years	*p*-value*
**MII**	38.4%	37.5%	28.6%	0.030
**Rescued MI-MII**	8.5%	9.7%	6.6%	0.128

* Kendall correlation

## DISCUSSION

In the present study, we evaluated whether rescue maturation after incubation of MI oocytes collected in COH cycles could be used for ICSI by evaluating the fertilization rate, polyploidy rate and blastocyst formation rate. We performed an early denudation to assess oocyte maturation, and from this, we were able to evaluate the maturation rate and consequent embryonic quality, since early denudation does not appear to compromise ICSE cycle outcomes (Naji *et al*., 2018). We found a rescued maturation rate of 57% of MI oocytes after 4 hours of incubation in culture medium, and these rates were similar to those of other studies that reported rates between 43 and 54% in the first 4-6 hours (Shu *et al*., 2007; Vanhoutte *et al*., 2005; Álvarez et al., 2013). Regarding incubation time, Strassburger *et al*. (2004) found that 13% of MI oocytes subjected to rescue maturation matured within 1 hour and that another 41% matured within 2.5 hours, revealing that most MI oocytes (54%) matured *in vitro* to MII oocytes after 2.5 hours of incubation; however, the performance of these oocytes with respect to embryonic development appears to be reduced compared with MII oocytes (Braga *et al*., 2020).

When evaluating the normal fertilization rate, we found that the MI oocytes that had not matured after 4 hours of incubation for rescue maturation had the lowest normal fertilization rate (31.9%) compared with oocytes of other stages, particularly MII oocytes (79.1%). We found that an MI oocyte was 7.6 times more likely to have fertilization failure than an MII oocyte. A low normal fertilization rate of immature oocytes has been reported by other studies, and among MI oocytes, the fertilization rate was lower than that of *in vitro*-matured MI oocytes (Alcoba *et al*., 2015; De Vincentiis *et al.*, 2013; Strassburger *et al*., 2004; 2010; Shu *et al*., 2007). The results of this study indicate that oocyte maturation has a direct influence on fertilization rates. The increased fertilization rate of MI-MII oocytes compared to immature MI oocytes seems to be related to the fact that they may not form meiotic spindles during cell division, they may exhibit failed cytoplasmic maturation, or they may fail to enter metaphase (Windt *et al*., 2001; Santiquet *et al*., 2017; Sen & Caiazza, 2013).

During their growth phase, oocytes accumulate proteins and RNAs to complete meiotic division and maintain cellular homeostasis. Any change in this process can lead to delayed and failed embryonic development (Jones *et al*., 2008). Studies evaluating MI-MII oocytes revealed that nuclear maturation occurs before the cytoplasm reaches full maturity, implying defects in their cytoplasmic and cytoskeletal organization, which alter the morphogenesis of the meiotic spindle and result in embryos with nuclear disorganization, chromosomal abnormalities, triploidy and polyploidy (Sanfins *et al*., 2004; Wang *et al*., 2001). Triploidy appears to be the most common abnormality and is characterized by the presence of three (3PN) instead of two (2PN) haploid chromosomal complements. During ICSI, which excludes the possibility of polyspermy, polyploidy can occur due to one of the following: fertilization of a haploid egg by a diploid sperm or fertilization of a diploid egg by a haploid sperm (Rosenbusch, 2008). We found a high polyploidy rate after ICSI with MI oocytes, with a 2.6-fold higher risk compared with MII oocytes. Polyploid embryo formation may indicate a dysfunctional oocyte, and high rates of polyploid embryo formation are associated with a lower quality embryo cohort with a higher risk of implantation failure and abortion (Figueira *et al*., 2011).

In our study, we noticed that the blastocyst formation rate was highest among embryos from MII oocytes (36.4%), that the blastocyst formation rate among embryos from MI-MII oocytes was 11.4%, and embryos from MI oocytes had the lowest rate, at 0.6%. Previous studies have demonstrated the influence of oocyte maturation on the rate of blastocyst formation. Immature oocytes, in addition to having a lower fertilization rate and lower blastocyst formation rate, have higher implantation failure and abortion rates (Kedem *et al*., 2018; Son *et al*., 2013; Zhao *et al*., 2009; Shu *et al*., 2007). De Vos *et al.* (1999) and Alcoba *et al*. (2015) did not evaluate the rate of blastocyst formation in their studies; they evaluated the cleavage rate in D2 and D3, and found that the cleavage rates were similar between embryos from MII oocytes and those from MI-MII oocytes, demonstrating that *in vitro*-matured oocytes also have the ability to generate good-quality embryos. However, we know that the evaluation of embryos in the cleavage stage does not represent the true embryonic capacity; and therefore, embryo cultivation to the blastocyst phase has become the most commonly used approach by most assisted reproduction centers (Alcoba *et al*., 2015; Piqueras *et al*., 2017). Thus, although the cleavage rate seems to be similar between embryos derived from *in vivo*-matured (MII) and *in vitro*-matured (MI-MII) oocytes, as described in the studies cited above, the results of our study demonstrate that the blastocyst formation rate from MI-MII oocytes is significantly lower than that from MII oocytes.

In addition, immature MI oocytes have a blastocyst formation rate of less than 1%, and thus, immature *in vitro* MI oocytes have very low developmental potential for progression to the blastocyst stage, and should not be used. Although this is one of the conclusions of this study, it is important to emphasize that the one blastocyst that did form from an immature MI oocyte generated a pregnancy that culminated in the birth of a healthy baby. For this couple who had only that particular embryo for transfer, the statistics on the rate of blastocyst formation were not relevant, as they managed to have a healthy baby even after the fertilization of an immature MI oocyte. In a review of the current literature, we found one publication on ICSI outcomes with MI oocytes that were injected without extrusion of their PB for a single embryo transfer. This embryo originated from one oocyte that had arrested at the MI stage during *in vitro* maturation, and this oocyte that was injected and fertilized, underwent cleavage, was transferred, had implanted and developed to a full-term pregnancy with a healthy neonate (Strassburger *et al*., 2004). Furthermore, the rates of normal fertilization (2PN) after ICSI of MI oocytes (25-58%) in previous reports are similar to our observed rate of 31.9% (Álvarez et al., 2013; Shu *et al*., 2007; Strassburger *et al.*, 2004; 2010). This demonstrates that the MI oocyte can complete the extrusion of its PB even after ICSI; however, the quality of embryo development may be low because of DNA disorganization. One study that examined the chromosomal content of embryos resulting from MI oocytes after COH demonstrated that 70.2% of embryos from rescued MI-MII oocytes and 97.2% from arrested MI are genetically abnormal, with 0% and 6.4% of them showing polyploidy, respectively, thus demonstrating that it is possible to form euploid embryos after ICSI of MI oocytes. However, in the aforementioned study, they analyzed probes for only three chromosomes and indicated that a complete chromosomal analysis may increase the rates of aneuploidy (Strassburger *et al*., 2010).

We also evaluated the ages of women in relation to the blastocyst formation rate. We found a decrease in the rate of blastocyst formation from MII oocytes in the age group of patients older than 40 years but no difference in the rate of blastocyst formation from MI-MII oocytes. The rate of blastocyst formation from MII oocytes was similar to that found in other studies (Macklon *et al*., 2002; Durand *et al*., 2016; Cimadomo *et al*., 2018), and the woman’s age was a major factor in the success of IVF due to the decreased oocyte quality, decreased blastocyst formation rate and increased risk of aneuploidy in this age group (Su *et al.*, 2017; Macklon *et al.*, 2002). However, maternal age does not seem to influence the rate of blastocyst formation from MI-MII oocytes, suggesting that they may be used for ICSI. The use of MI-MII oocytes may be of particular importance in women with low ovarian reserve because, despite having a lower fertilization rate and a lower blastocyst formation rate than MII oocytes, MI-MII oocytes are still capable of generating a good blastocyst for transfer. On the other hand, MI oocytes that have not rescue matured in vitro to MII oocytes should not be used, as the fertilization rate is low, with a high polyploidy rate, and especially because the rate of blastocyst formation is approximately zero, with a high risk of embryonic aneuploidy.

## CONCLUSION

In summary, in the present study, we found that oocytes collected at the MI stage after COH that do not rescue mature in vitro to MII oocytes have a reduced fertilization rate, a high incidence of embryo polyploidy and a blastocyst formation rate of only 0.6%. Therefore, the use of immature MI oocytes is not justified and should be discouraged. On the other hand, although the blastocyst formation rate from rescue in vitro-matured MI-MII oocytes has been observed to be significantly lower than that from MII oocytes, the use of MI-MII oocytes should be encouraged, especially in women with a low MII oocyte yield, as these oocytes may increase the number of blastocysts available for embryo transfer.
